# Structure parameter design and bench test research of paddy field blades

**DOI:** 10.1038/s41598-022-19115-6

**Published:** 2022-08-30

**Authors:** Chongcheng Chen, Zhifeng Di, Weixiang Chen, Shuhe Zheng, Jinbo Ren

**Affiliations:** 1 College of Mechanical and Electrical Engineering, Fujian Agriculture and Forestry University, Fuzhou, 350002 China; 2 Shandong Academy of Agricultural Machinery Sciences, Shandong, 250103 China

**Keywords:** Mechanical engineering, Engineering

## Abstract

Aiming at the problems of high labor intensity and low efficiency in manual operation during the pulping process of rice seedling nursing in thin mud in hilly and mountain areas, this paper designed a new type of paddy field blade for seedling nursing, and utilized a rice nursery pulper to help manual operation complete the pulping process. It created a mathematical model of mud-throwing mass and operating power in the operation process of paddy field blade, and obtained the main structure factors that influenced the mud-throwing mass and operating power of paddy field blade, which included the area of mud splashing board, the angle between the front cutting edge and the mud splashing board, and the inclination angle between the front cutting surface and the mud splashing board. To further analyze the degree of influence of the blade’s main structure parameters on mud-throwing mass and operating power, it used EDEM discrete element simulation software to establish a discrete element simulation model of paddy field blade and mud particle system, performed simulation analysis with the method of orthogonal experimental design, and conducted a bench test for comparison. The results showed that: (1) the degree of influence of the three structure parameters on mud-throwing mass and operating power from large to small in order was the area of mud splashing board > the inclination angle between front cutting surface and mud splashing board > the angle between front cutting edge and mud splashing board; (2) the maximum relative error for mud-throwing mass between simulation analysis and bench test was 4.53%, and that for operating power was 8.67%; and (3) three reference parameters combinations were selected by P_2_-1/M_2_ graph, the mud-throwing mass of the three combinations was 40.43%, 27.52% and 0.16% higher than that of the original blade, and the power consumed was 13.99%, 21.83% and 36.65% lower than that of the original blade, indicating that the new paddy field blade had good operating performance.

## Introduction

**Figure 1:**
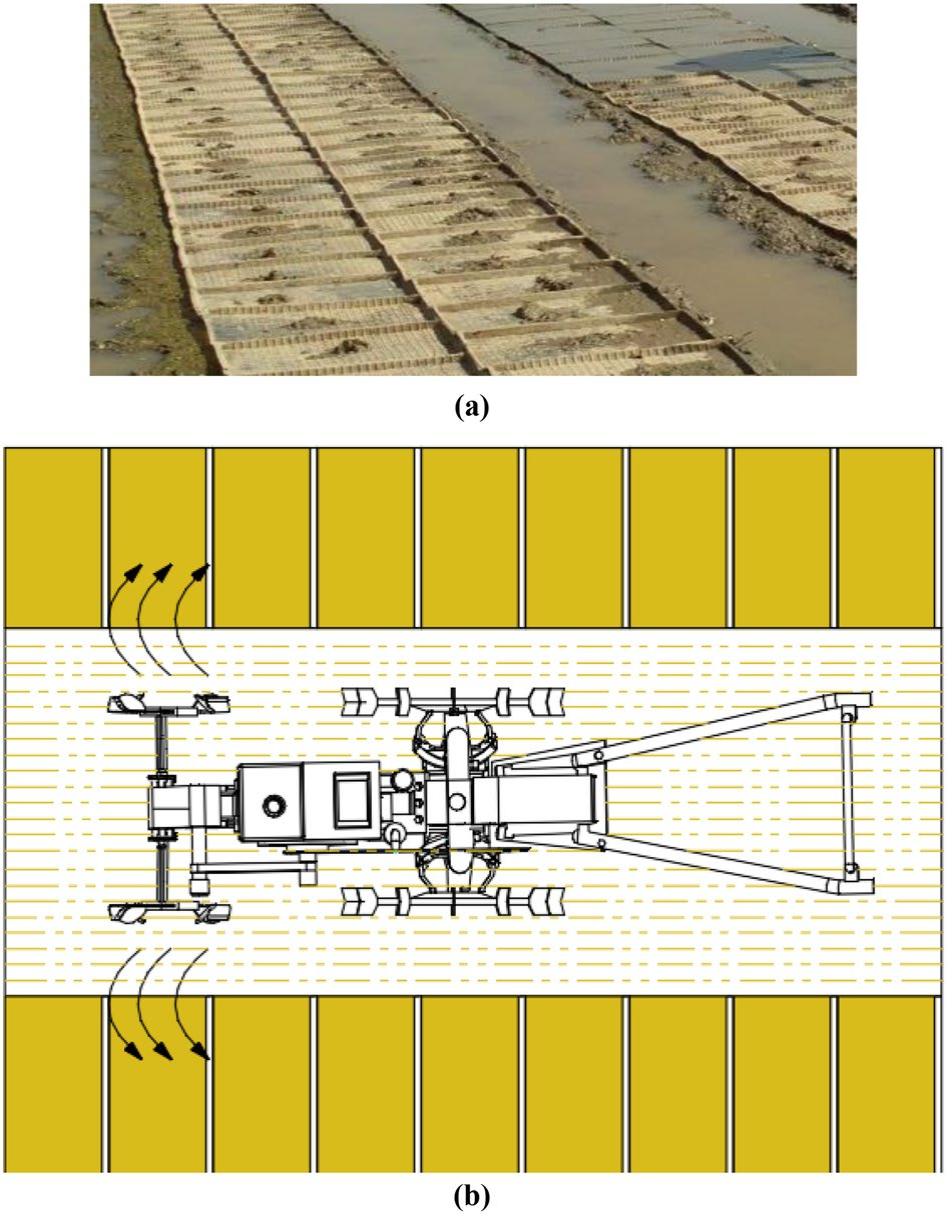
Working background of the paddy field pulper. (**a**) Rice nursery environment. (**b**) Simple diagram of paddy field pulper operation.

Rice is one of the most important food crops worldwide. Many countries such as the United States and Australia primarily adopt a direct seeding method for its production as it is easy and convenient^1^. Owing to the unique topography and climate in Asia, direct seeding rice has a low survival rate. Therefore, in order to ensure a high survival rate of rice seedling, transplanting planting mode with mud seedling trays is often used in the rice cultivation process^2^. Rice transplanting equipment has successively emerged since the 1980s. As for the mud used in rice seedling nurseries, currently most areas still use manual pulping, which has the shortage of high labor intensity and low efficiency. To address this issue, scholars around the world have conducted many studies. For instance, Zhang Shubiao et al.^3^ designed a manual type of mud filter that could chop mud blocks manually; Pang Changle et al.^4^ designed a mud laying device that could achieve continuous indoor laying of mud, but it could not prepare rice seeding mud; Guo Hongjiang et al.^5^ designed a rice field seedling laying machine that could stir and chop mud-mixed soil blocks poured into the funnel, but it still required a lot of human labour in the actual application; and Sun Guodong et al.^6,7^ designed a mud lifting device that could directly transport the mud in a paddy field to a certain height through a spiral pipeline, but it had some limitations because it was fixed at a position in the real production process. At an earlier stage, the project team^8–13^ designed a machine used for pulping rice seedlings with thin mud. The working principle of this machine was similar to a rotary cultivator. While going forward in a paddy field, it used a paddy field blade to throw the mud in the paddy field into the rice seeding trays placed in its opposite side, as shown in Fig. 1. This study is supported by lots of rotary cultivator theories^14–16^, so it is easy to implement, and also it is expected to be the key to solving this issue in the future. However, relevant studies have not constructed a theoretical model for paddy field blade mud-throwing operation, and the working result of the original paddy field blade is not ideal. Thus, solving the problem of the blade can indirectly solve the issue of the low level of mechanization in rice seedling cultivation and pulping.

A right-angle blade was designed in Europe in the twentieth century and then it was widely used in dry land. Afterwards Japanese scholars improved the right-angle blade, designed a billhook^17^ and applied it in rice fields. On this basis, scholars in Asia have carried out a large number of research on power consumption^18,19^, incision curve^20^ and front cutting surface shape^18^ of rotary blade. With the wide application of rotary blade, various countries in the world specify their own standards for the rotary blade. This paper performed design and transformation based on the prototype of Chinese rotary blade. Firstly, it designed a new type of paddy field blade with better working results and analyzed the force situation of paddy field blade in the operation process. Then it constructed a theoretical model for mud-throwing mass and operating power of paddy field blade and adopted discrete element software to perform simulation analysis. Finally, it carried out a bench test to verify the reliability of the model and simulation results.

## Paddy field blade structure

**Figure 2:**
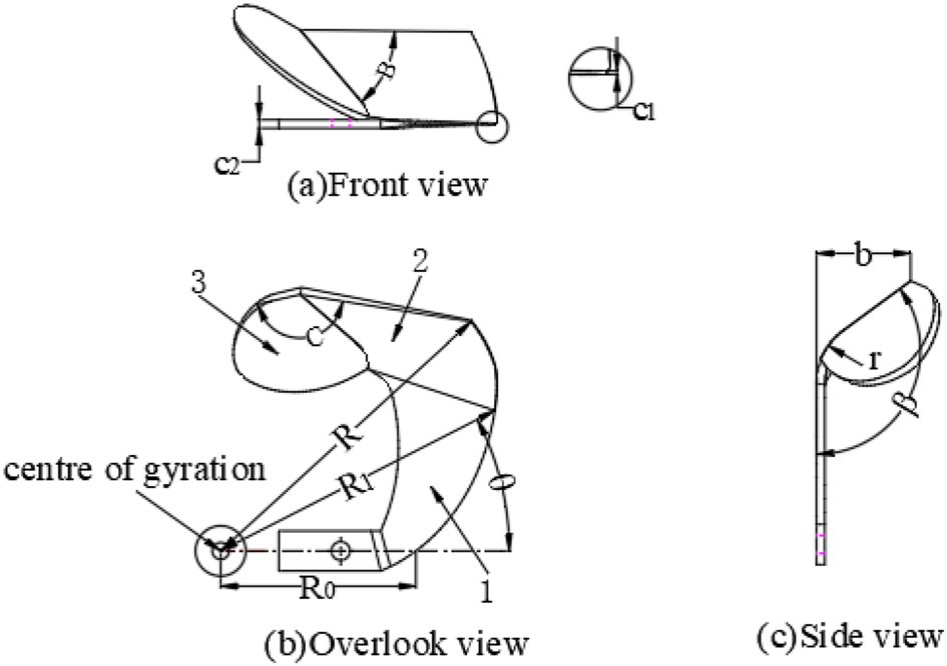
Paddy field blade structure. 1. Side cutting surface; 2. Front cutting surface; 3. Mud splashing board. B is the angle between the front cutting edge and the mud splashing board (°), C is the angle between the front cutting surface and the mud splashing board (°), R_0_ is the starting radius of the side cutting edge (mm), R_1_ is the ending radius of the side cutting edge (mm), R is the radius of gyration of the knife roll (mm), θ is the wrap angle of the side cutting edge (°), c1 is the thickness of the cutting edge (mm), c2 is the thickness of the knife handle (mm), b is the working width (mm), r is the bending radius (mm), and β is the bending angle of the front cutting surface (°).

The shape of the blade used for mud-throwing thin-mud rice seedlings is shown in Fig. 2. The blade primarily comprises a side cutting surface, side cutting edge, front cutting surface, front cutting edge, and mud splashing board. The side cutting edge adopts the same Archimedes curve as the rotary tiller, with the starting radius R_0_ = 100 mm, ending radius R_1_ = 170 mm, wrap angle θ = 30°, thickness of blade c2 = 5 mm, thickness of blade c1 = 1 mm, bending radius at the front cutting surface and side cutting edge r = 30 mm, bending angle β = 120°, blade working width b = 35 mm, and turning radius R = 200 mm.

## Mud-throwing performance

### Mud-throwing mass

**Figure 3:**
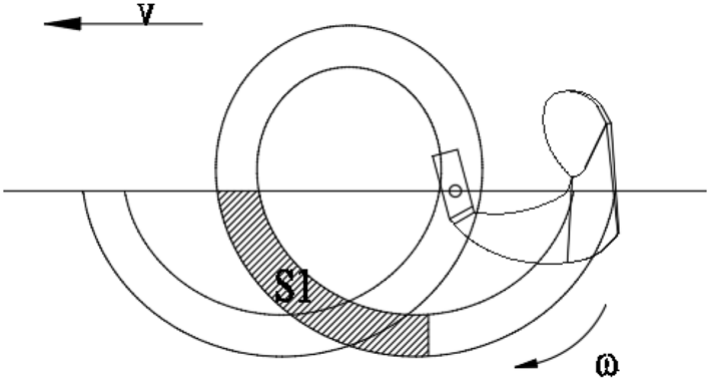
Blade movement trajectory. V is the speed of moving forward (mm/s), ω is the blade rotation speed (rad/s), and S1 is the volume of mud thrown (mm^3^).

During the working process, the blade performs a rotary motion to cut and throw mud, and it simultaneously moves forward with the whole machine. The actual motion is a superposition of rotary motion and linear motion, and its trajectory is a trochoidal line^21^, as shown in Fig. 3.

The mud-throwing mass during one rotation of the blade is:

1 $${M}_{pao}=A\cdot \mathit{sin}C\cdot \mathit{sin}B\cdot l\cdot \rho $$

where ρ is the mud density, with a value of 1530 kg/m^2^, A is the area of the mud splashing board, B is the angle between the front cutting blade and the mud splashing board, C is the angle between the mud splashing board and the front cutting surface, and $\mathrm{l}$ is the cutting length of the blade during the upward throwing.

2 $$l={\int }_{\frac{2\pi }{\omega }}^{\frac{3\pi }{2\omega }}\sqrt{{v}^{2}-2\cdot \omega \cdot v\cdot R\cdot \mathit{sin}\omega t+{R}^{2}\cdot {\omega }^{2}} dt$$

Considering that part of the mud will not be used after being thrown out, the mud-throwing efficiency coefficient k was introduced, and its value was determined to be 0.5–0.7 through experiments. The total mass of the mud thrown during a certain period of blade operation is:

3 $${M}_{1}=2\cdot k\cdot \frac{\omega }{\pi }\cdot t\cdot {M}_{pao}$$

where t is the operating time (s) and ω is the rotational angular velocity (rad/s).

### Operating power

#### Blade force analysis

**Figure 4:**
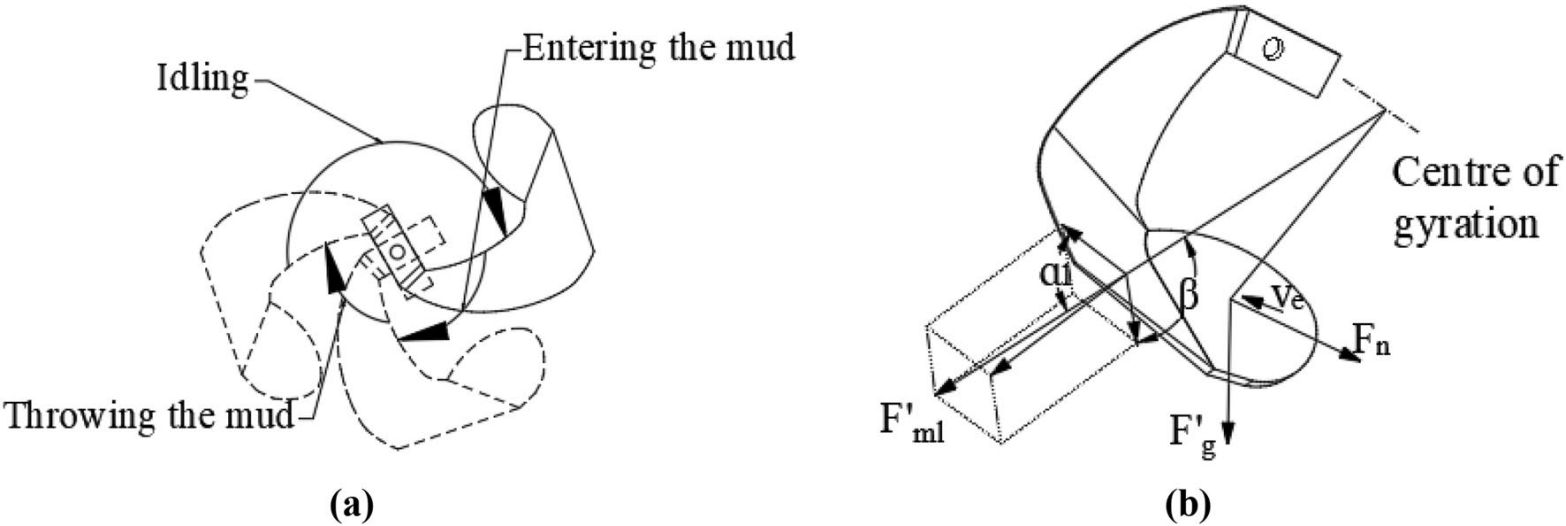
Operation process and force diagram of paddy field blade (**a**) operation process; (**b**) force analysis. ɑ1 is the angle between the front cutting surface and the velocity direction (°), β is the bending angle of the tangent plane (°), ${\mathrm{F{^{\prime}}}}_{\mathrm{ml}}$ is the centrifugal force of the mud (N), F'g is the gravitational force of the mud (N), Fn is the viscous force of the mud (N), and Ve is the absolute speed direction of the blade.

The operation of a single blade on the paddy tool can be divided into three processes—idling, entering the mud, and throwing the mud—as shown in Fig. 4. It is assumed that each blade is continuously cycling through these three processes during the operation.

1. Force during idling process
  The idling process is when the blade is rotating in the air and not touching the mud. The angle of the tool rotation is between 0° and 180°, and the tool is affected by its own gravity and centrifugal force:
  4 $$\overrightarrow{F}={\overrightarrow{F}}_{g}+{\overrightarrow{F}}_{l}$$
  where ${\overrightarrow{\mathrm{F}}}_{\mathrm{g}}$ is the gravitational force of the blade and ${\overrightarrow{\mathrm{F}}}_{\mathrm{l}}$ is the centrifugal force.
2. Force during mud entering process
  The process during which the blade cuts into the mud from the beginning to move to the lowest point is called the process of entering the mud, and the rotation angle of the tool in this process is between 180° and 270°, with the blade being affected by the gravitational, centrifugal, driving, and viscous forces (Newtonian friction force) of the mud^14^:
  5 $$\overrightarrow{F}={\overrightarrow{F}}_{mz}+{\overrightarrow{F}}_{l}+{\overrightarrow{F}}_{g}+{\overrightarrow{F}}_{ml}+{\overrightarrow{F}}_{n}$$
  where ${\overrightarrow{\mathrm{F}}}_{\mathrm{mz}}$ is the driving force of the mud, ${\overrightarrow{\mathrm{F}}}_{\mathrm{l}}$ is the centrifugal force of the blade, ${\overrightarrow{\mathrm{F}}}_{\mathrm{g}}$ is the gravitational force of the blade, ${\overrightarrow{\mathrm{F}}}_{\mathrm{ml}}$ is the centrifugal force of the mud, and ${\overrightarrow{\mathrm{F}}}_{\mathrm{n}}$ is the viscous force of the mud.
  When the mud rotates at a uniform speed, the centrifugal force that the blade needs to provide is ${\mathrm{F{^{\prime}}}}_{\mathrm{ml}}$. After force analysis, the component force of centrifugal force in the direction of velocity is determined as follows:
  6 $${F}_{ml}=4{M}_{pao}\cdot {\omega }^{2}\cdot R\cdot \mathrm{sin}{\alpha }_{1}\cdot {\mathrm{sin}}^{2}\beta \cdot \mathrm{cos}{\alpha }_{1}$$
  Paddy mud is a Bingham fluid, and its viscous force is:
  7 $${F}_{n}=(\mu \frac{dv}{dh}+{\tau }_{0})\cdot A$$
  where $\frac{\mathrm{dv}}{\mathrm{dh}}$ is the shear rate, µ is the viscosity of the mud, ${\uptau }_{0}$ is the yield strength of the mud, and A is the contact area between the blade and mud (the blade has two sides; hence, this is twice the area of the blade).
3. Force during mud-throwing process

The process during which the blade comes up from the lowest point to the surface of the mud is called the mud-throwing process. The rotation angle of the tool at this stage is between 270° and 360°. In addition to the blade’s own weight and centrifugal force, the main forces at this stage are the gravitational and centrifugal forces of the mud after uniform rotation, and the viscous force of the mud hindering the movement of the blade:

8 $$\overrightarrow{F}={\overrightarrow{F}}_{mg}+{\overrightarrow{F}}_{l}+{\overrightarrow{F}}_{g}+{\overrightarrow{F}}_{ml}+{\overrightarrow{F}}_{n}$$

where ${\overrightarrow{\mathrm{F}}}_{\mathrm{mg}}$ is the gravitational force of the mud, ${\overrightarrow{\mathrm{F}}}_{\mathrm{l}}$ is the centrifugal force of the blade, ${\overrightarrow{\mathrm{F}}}_{\mathrm{g}}$ is the gravitational force of the blade, ${\overrightarrow{\mathrm{F}}}_{\mathrm{ml}}$ is the centrifugal force of the mud, and ${\overrightarrow{\mathrm{F}}}_{\mathrm{n}}$ is the viscous force of the mud.

#### Power analysis

As the blade completes one revolution, the gravitational and centrifugal forces do zero work, that is:

9 $${\mathrm{W}}_{\mathrm{g}}=0 {\mathrm{W}}_{\mathrm{l}}=0$$

The work done by the resistance of the blade entering the mud can be approximately converted into the kinetic energy throwing the mud. At the same time, the work done by the blade against gravity is:

10 $${W}_{mz}=2{M}_{pao}\cdot {\omega }^{2}\cdot {R}^{2}$$

11 $${W}_{mg}={4M}_{pao}\cdot g\cdot R$$

where g is the acceleration due to gravity.

The total work done by a single blade in one revolution is:

12 $$W={W}_{mz}+{F}_{ml}\cdot 2l+{W}_{mg}+{F}_{n}\cdot 2l$$

The average operating power of the entire tool is:

13 $${P}_{1}=\frac{W}{t}$$

where t is the time required for the tool to complete one revolution.

## Virtual simulation test

### Experimental design

**Figure 5:**
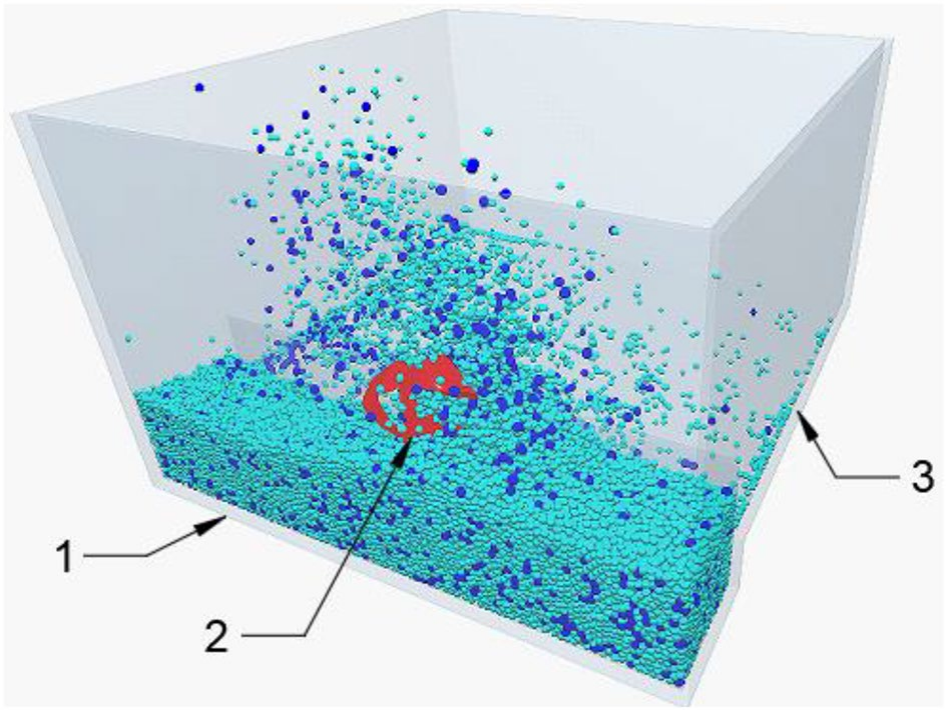
Simulation test environment. 1. Mud pool 2. Tool 3. Collection tray.

It can be known from theories on paddy field blades that parameters such as the area of mud splashing board, the inclination angle between front cutting surface and mud splashing board, and the angle between front cutting edge and mud splashing board are the main factors that affect mud-throwing mass and operating power. According to the actual production and the agronomic needs of seedling nursing, mud-throwing mass and operating power are chosen as technical evaluation parameters. EDEM simulation software is used to perform orthogonal experimental design^22^. The simulation tool is drawn in the Solid Works software, and the simulation test process is shown in Fig. 5.

L16(4^3^)orthogonal experimental method is used. This orthogonal experiment is able to design a freer factor level table^23,24^. It can not only meet the situation that the factor level of blade is uncertain at an earlier design stage, but also verify the mathematical model and select the parameters combinations. The factor level table of experiment design is shown in Table 1.

**Table 1:** Factor level table.

Level	Factor		
	A, Splash plate area (mm^2^)	B, Tangent blade-splash plate angle (°)	C, Tangent plane-splash plate angle (°)
1	3000	45	110
2	3500	50	120
3	4000	55	130
4	4500	60	140

### Analysis of simulation results

#### Range analysis

The test arrangement and results are shown in Table 2, which indicates the following. (1) The range R of the mud splashing board area with respect to the mass of mud thrown and power is the largest, indicating that the mud splashing board area has the greatest impact on the mass of mud thrown and operating power of the blade; the mass of mud thrown and working power increase with the area. (2) The angle between the front cutting edge and the mud splashing board has the least influence on the blade. The larger the knife angle, the greater the mud-throwing mass and power used, and when the angle is between 50° and 55°, the mud-throwing mass increases significantly, whereas the changes between other angles are relatively small, similar to the changes in the operating power. (3) The larger the angle between the front cutting surface and the mud splashing board, the lower the mud-throwing mass and operating power. When the angle is between 110° and 120°, the change in power is relatively slow; conversely, the power decreases rapidly between 120° and 140°, and the slurry amount changes almost linearly. (4) The influence of various factors on the mud-throwing mass, from high to low, is as follows: area of mud splashing board > angle between front cutting surface and mud splashing board > angle between front cutting edge and mud splashing board.

**Table 2:** Experimental method and results.

Blade number		Factor level					Mud-throwing mass M_2_(kg)	Power, P_2_ (W)
		A, Level of splash plate area		B, Level of tangent blade-splash plate angle		C, Level of Tangent plane-splash plate angle		
1		1		1		1	16.26	315.65
2		1		2		2	16.72	326.87
3		1		3		3	16.85	322.07
4		1		4		4	15.75	313.97
5		2		1		2	17.83	349.32
6		2		2		1	18.87	361.47
7		2		3		4	17.36	330.52
8		2		4		3	18.55	376.45
9		3		1		3	17.62	339.20
10		3		2		4	17.03	343.07
11		3		3		1	21.41	412.22
12		3		4		2	21.21	404.02
13		4		1		4	16.70	322.40
14		4		2		3	18.15	355.70
15		4		3		2	21.40	416.50
16		4		4		1	25.30	452.42
M_2_	K1	65.58		68.41		81.84		
	K2	72.61		70.77		77.16		
	K3	77.27		77.02		71.17		
	K4	81.55		80.81		66.84		
	R	15.97		12.4		15		
P_2_	K1	1278.575		1326.575		1541.775		
	K2	1417.775		1387.125		1496.725		
	K3	1498.525		1481.325		1393.425		
	K4	1547.025		1546.875		1309.975		
	R	268.45		220.3		231.8		

M_2_ is mud-throwing mass and P_2_ is power obtained in the experiment.

#### Analysis of variance

The extent of influence each factor has on the target was determined. The variance analysis method was applied to analyze the orthogonal experimental data, M_2_ and P_2_ was used to measure the significance of the relationship that each factor has to the target. The analysis of variance calculation is shown in Table 3.

**Table 3:** Analysis of variance.

Target	Source	df	Seq SS	Adj SS	Adj MS	F	P	Sig
M_2_	A	3	35.067	35.067	11.6891	13.65	0.004	***
	B	3	24.231	24.231	8.0769	9.43	0.011	**
	C	3	32.618	32.618	10.8726	12.69	0.005	***
	Error	6	5.14	5.14	0.8566			
	Total	15	97.055					
P_2_	A	3	10,337.4	10,337.4	3445.8	27.55	0.001	***
	B	3	7177.3	7177.3	2392.4	19.13	0.002	***
	C	3	8142.4	8142.4	2714.1	21.7	0.001	***
	Error	6	750.3	750.3	125.1			
	Total	15	26,407.5					

*Relatively significant (P < 0.10); **significant (P < 0.05); and ***extremely significant (P < 0.01).

The analysis of variance between the mount of mud thrown and operating power shows the following. (1) The mud splashing board area, the angle of the front cutting edge, and the inclination angle of the mud splashing board have a significant impact on the mass of mud thrown; the blade angle of the front surface has a significant impact on the mass of mud thrown. (2) The influence of each factor on the operating power is extremely significant. (3) The following is the factor ranking in order of influence significance: mud splashing board area > angle between front cutting surface and mud splashing board > angle between front cutting edge and mud splashing board; this is consistent with the results of range analysis.

#### Parameter selection

To determine the working parameter combination of blade operation, the results of the test was analyses, and the principle of increasing the mass of mud thrown and reducing the working power was set. Combining the boundary conditions of the test factors, the parameter model of nonlinear programming was established as follows:

14 $$\left\{\begin{array}{c}max {M}_{2}\\ min {P}_{2}\\ s.t\left\{\begin{array}{c}3000{\mathrm{ mm}}^{2}\le A\le 4500{\mathrm{ mm}}^{2}\\ 45^\circ \le B\le 60^\circ \\ 110^\circ \le C\le 140^\circ \end{array}\right.\end{array}\right.$$

**Figure 6:**
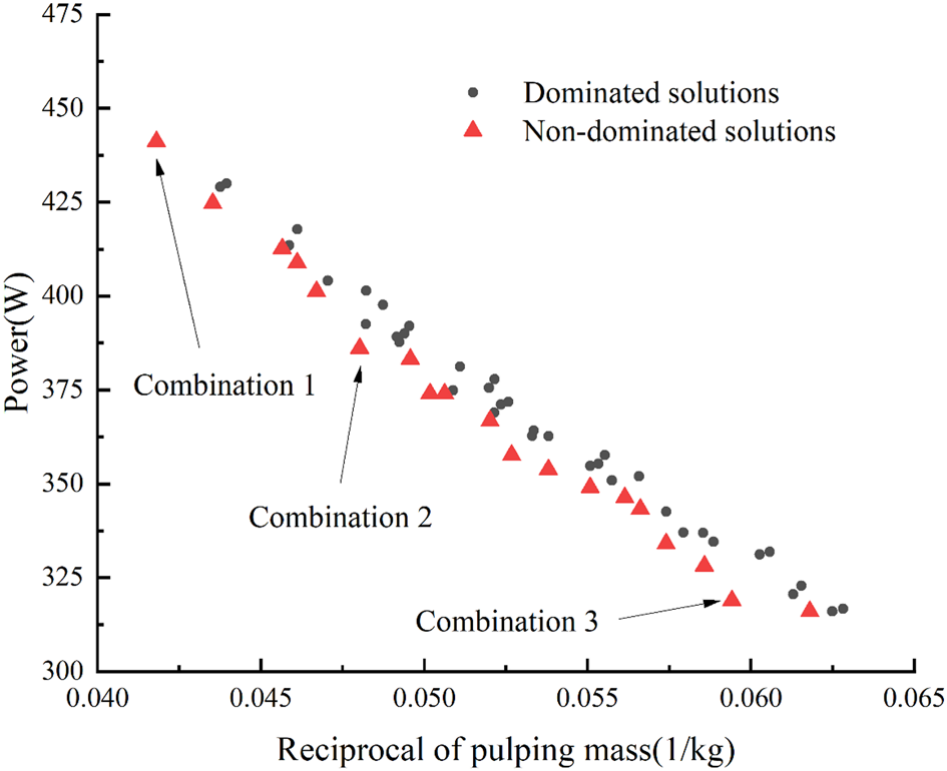
P_2_-1/M_2_ graph.

Design-Expert is a kind of experiment design software developed by Stat-Ease, an American company, and it has been widely used in design and analysis of multifactor experiments^25^. Design-Expert8.0.5b software was used in this paper to fit, analyze and solve the parameter model and get the combination data that were not obtained in the orthogonal experiment. P_2_-1/M_2_ graph was drawn for all combinations, as shown in Fig. 6. After screening, a total of 19 combinations with Pareto frontier were obtained. As the actual production requires different weights of power and mud-throwing mass, the combinations selected should be also changed. Here three combinations that may be common in the actual production were listed for reference. Combination 1 can be chosen when paying more attention to mud-throwing efficiency and less attention to power consumption, in which the area of mud splashing board is 4500 mm^2^, the angle between front cutting edge and mud splashing board is 60°, the inclination angle between front cutting surface and mud splashing board is 110°, the mud-throwing mass is 23.924 kg, and the operating power is 441.181 W. Combination 2 can be chosen when paying equal attention to mud-throwing mass and power, in which the area of mud splashing board is 4500 mm^2^, the angle between front cutting edge and mud splashing board is 45°, the inclination angle between front cutting surface and mud splashing board is 110°, the mud-throwing mass is 20.82 kg, and the operating power is 386.10 W. Combination 3 can be chosen when paying more attention to power consumption and less attention to mud-throwing mass, in which the area of mud splashing board is 3000 mm^2^, the angle between front cutting edge and mud splashing board is 45°, the inclination angle between front cutting surface and mud splashing board is 110°, the mud-throwing mass is 16.832 kg, and the operating power is 318.994 W.

### Comparative analysis of model and simulation

**Figure 7:**
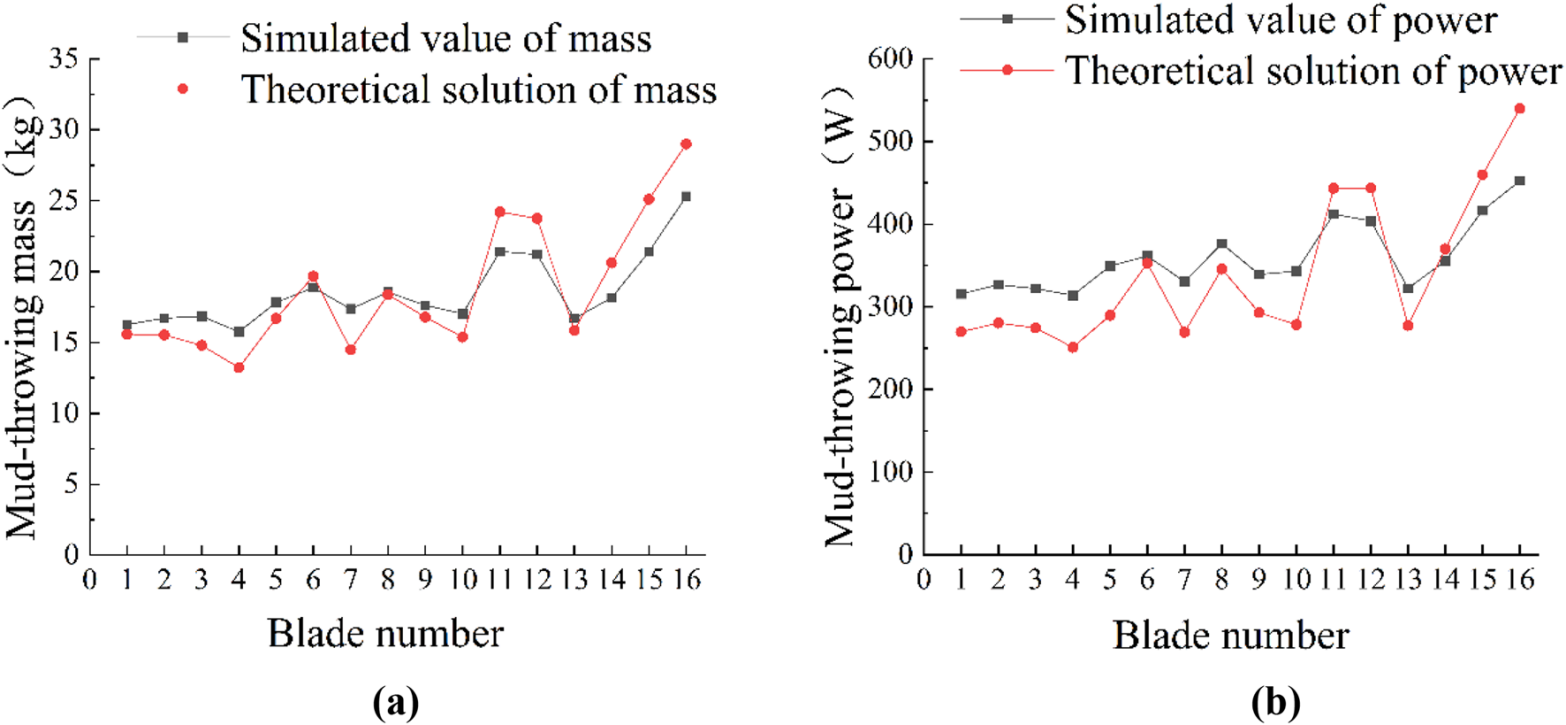
Trend comparison chart. (**a**) Comparison of mud quantity trends; (**b**) comparison of operating power trends.

MATLAB 2018b was used to calculate the mass and power numerical solution of the paddy field blade mud-throwing operation model and compare it with the simulation results, as shown in Fig. 7. It can be seen from Fig. 7 that when both mud-throwing mass and power of blade are high, the model results are higher than the simulation results, probably because the problem of mud backflow is not considered in the model. The figure indicates that the maximum relative error between the theoretical solution and the simulated value of the mud mass is 9.95%, and the maximum relative error between the theoretical solution of simulated value of power is 13.5%, which are within the allowable range of error. There is also a certain similarity between the theoretical model and the simulation data of blades with different structural parameters, which shows that the established simulation model can simulate the process of mud-throwing relatively accurately.

## Bench test

**Figure 8:**
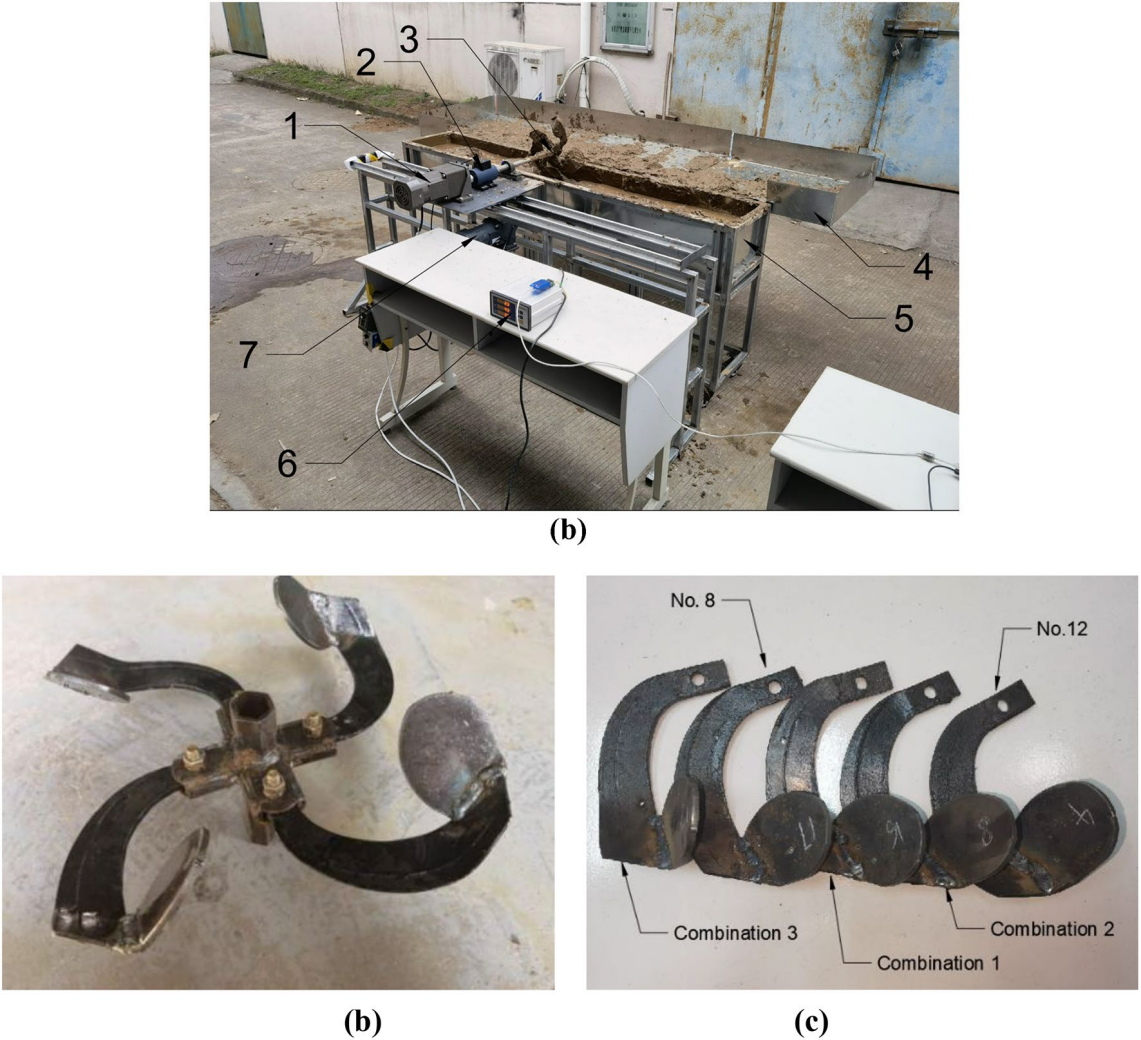
Bench test. (**a**) Bench construction 1. Geared motor 2. Torque speed sensor 3. Blades 4. Collecting tray 5. Mud pool 6. Power analyzer 7. Asynchronous motor; (**b**) experiment tools; (**c**) blade number.

Bench test was performed in the agricultural engineering laboratory of Fujian Agriculture and Forestry University. The bench test device is shown in Fig. 8, and it includes mud pool, power analyzer LDN-08D (Beijing Longdingjinlu Measurement and Control Technology Co., Ltd., China), asynchronous motor YL90S-2 (Shanghai Han’ao Electric Co., Ltd., China), CBX1204-1000 geared motor, rack and pinion sliding table guide rail, paddy field blade, variable-frequence governor, catch tray and H8C-C3-1.5T-4B weighing sensor (ZEMIC, America). Blades 8 and 12 (control experiments) and three reference combination of blades in the simulation experiment were selected. A total of five sets of tools were processed, each with four blades.

During the experiment, the geared motor drove the blade to rotate to throw the mud in the paddy field into the catch tray on one side. Meanwhile, the asynchronous motor drove the blade forward through the rack and pinion mechanism to simulate the advance of the pulper in the paddy field. Preliminary experiments were performed before each experiment, and the parameters of electric machine were adjusted to stabilize the rotating speed of the blade at 25 rad/s and the forward speed of the blade at 0.5 m/s. The parameters of electric machine in each experiment were set according to the preliminary experiments, so as to keep the rotating and forward speeds of the blade unchanged. After stable operation of the blade, the power analyzer was used to analyze and record the operating power of the blade. Stopped operation after stable operation of the blade for 4 s, and then the weighing sensor was used to measure the mud mass in the catch tray, which served as mud-throwing mass. Each group of tools was tested thrice, and the results were their average value. The relative error was obtained by combining the results of virtual simulation experiment to verify the reliability of virtual simulation experiment. The experimental results are shown in Table 4.

**Table 4:** Bench test results.

Blade number	Experimental value		Stimulation value		Relative error/%	
	Mud-throwing mass/kg	Power/W	Mud-throwing mass/kg	Power/W	Mud-throwing mass	Power
Blade 8	19.43	399.02	18.55	376.45	4.52	5.65
Blade 12	20.39	442.38	21.21	404.02	4.02	8.67

Table 4 shows that, in general, the power of the paddy field blade in operation is slightly higher than the simulated power; this may be because the acceleration period at the beginning of blade operation was not considered in the simulation stage. The mass of mud thrown was very close. Among them, there was a slight difference in mud-throwing mass of Blade 8 and 12 between the experimental value and the stimulation value, and this was caused by the difference in the position of the catch tray during the experiment and simulation. The relative errors of the two groups of bench test and simulation experiment were 4.53% and 4.02% for mud-throwing mass, 5.65% and 8.67% for operating power. Therefore, the relative errors of all the experiments were between 3 and 10%, indicating that the simulation results using EDEM were reliable.

Under the same condition, bench test was also performed for the paddy field blade used by the team at an earlier stage in literature^12,13^. The results of three blade combinations were compared with the blade at an earlier stage. The experimental results are shown in Table 5.

**Table 5:** Results of comparative experiments.

Paddy field blade	Mud-throwing mass/kg	Change rate of mud-throwing mass (%)	Power/W	Change rate of power reduction (%)
Original Blade	16.494	–	499.201	–
Combination 1	23.164	40.43	429.324	13.99
Combination 2	21.035	27.52	390.250	21.83
Combination 3	16.520	0.16	316.239	36.65

It can be observed from Table 5 that by comparison, the mud-throwing mass of the three reference combinations was 40.43%, 27.52% and 0.16% higher than that of the original blade, and the power consumed was 13.99%, 21.83% and 36.65% lower than that of the original blade, indicating that the new blade can increase mud-throwing mass and meanwhile decrease operating power significantly. Thus, the new paddy field blade is obviously better than that of original blade in bench tests.

## Conclusion

Aimed at addressing the issue of low pulping efficiency during rice seeding, this paper proposes a new type of paddy blade with discrete element simulation experiments. A bench test was carried out to verify the reliability of the relevant simulation results. The main conclusions are as follows:

1. According to the working state analysis of the paddy field blade, three geometric parameters that have a greater impact on operation were selected and orthogonal experiments were carried out. Keeping the physical parameters unchanged, the degree of influence of the three parameters on the mud-throwing mass and the operating power, from large to small, was as follows: area of mud splashing board > angle between front cutting surface and mud splashing board > angle between front cutting edge and mud splashing board.
2. Two groups of paddy field blades were selected to perform a bench test, and it was compared with simulation analysis. The results showed that the relative errors were 4.53% and 4.02% for mud-throwing mass, 5.65% and 8.67% for operating power, which are within the acceptable range, indicating that the simulation model were accurate and reliable, and discrete element simulation can be used to effectively simulate the working performance of paddy field blade.
3. With the orthogonal experimental three reference combinations of paddy field blade were obtained. By comparing the working performance of paddy field blade in the three parameter combinations with that of original blade, the mud-throwing mass was 40.43%, 27.52% and 0.16% higher than that of the original blade, and the power consumed was 13.99%, 21.83% and 36.65% lower than that of the original blade, indicating that the working performance of new paddy field blade is obviously better than that of original blade. Different blade parameter combinations can be chosen according to different needs in actual production, which can provide a reference for practical production and operation of paddy field blade.
